# Arabidopsis accessions and their difference in heat tolerance during meiosis

**DOI:** 10.1093/plphys/kiaf055

**Published:** 2025-02-12

**Authors:** Joke De Jaeger-Braet

**Affiliations:** Assistant Features Editor, Plant Physiology, American Society of Plant Biologists; IST Austria (Institute of Science and Technology Austria), Klosterneuburg 3400, Austria

With the changing climate, it is becoming more and more crucial to understand the natural existing heat tolerance in plants for agricultural and breeding purposes. The model plant *Arabidopsis thaliana* is well suited for the study of natural genetic variation with its many sequenced accessions (Lian et al. 2024). In this issue of *Plant Physiology*, Zhao and colleagues analyzed and compared 2 of those accessions, Columbia (Col-0) and Landsberg *erecta* (L*er*), and their hybrids to understand the mechanisms behind meiotic heat tolerance (Zhao et al. 2024).

Meiosis is a specialized cell division that is an essential part of sexual reproduction. In meiosis, 1 round of DNA replication is followed by 2 rounds of chromosome segregation events, halving the chromosome number. In addition to random chromosome segregation, genetic diversity of the progeny occurs through the exchange of genetic information between homologous chromosomes (homologs) through a process called meiotic recombination (Zickler and Kleckner 2023). Meiotic recombination is initiated by the formation of double strand breaks (DSBs) in DNA. Those DSBs get repaired by DNA strand insertion into the homolog, which leads to the formation of cross-overs (COs).

Although the effect of high temperature on meiotic division has been studied over the last decades (reviewed in De Jaeger-Braet and Schnittger 2024), a detailed molecular understanding of it is still lacking. Previously, Zhao and colleagues have investigated the molecular impact of extreme heat stress during meiosis (Ning et al. 2021; Fu et al. 2022; Zhao et al. 2023). Here, they continued their investigation by making use of previous studies that showed that there are natural genetic differences in stress tolerance and meiotic recombination (Sidhu et al. 2015; Ziolkowski et al. 2017; Chen et al. 2021; Zhu et al. 2021).

Zhao et al. investigated the effect of extreme heat stress (37 °C) on meiosis in Col-0 and L*er* plants by first looking at the end product of meiosis, 4 haploid cells that are also called tetrads. Under nonstressed conditions, both Col-0 and L*er* form 4 equally sized cells, whereas under heat stress irregular meiotic end products could be found. The irregularities include unequally sized cells and deviating number of cells, that is, 2, 3, or more than 4. The occurrence of those irregular products was much more frequent in Col-0 than in L*er*, 86% and 9%, respectively. In addition, this difference was also reflected in pollen viability defects between Col-0 and L*er* that indicate that the L*er* accession is more thermotolerant than Col-0.

To further investigate this higher thermotolerance of L*er*, Zhao et al. surveyed different steps of the meiotic division: microtubule organization, DSB and CO formation, pairing of homologs, and synapsis (the stabilization of paired homologs). Comparing the results of those different aspects between L*er* and Col-0 plants under heat stress led to the conclusion that the meiotic division of L*er* is less affected by heat compared with Col-0 (Fig. A). Specifically, heat does not affect synapsis and recombination in L*er.* For example, under nonstressed conditions at the end of the recombination pathway, around 11 and 9 COs are made in Col-0 and L*er*, respectively (Ziolkowski et al. 2017). Under extreme heat stress, no COs could be detected in Col-0, in contrast to L*er*, which showed around 10 COs.

**Figure.**
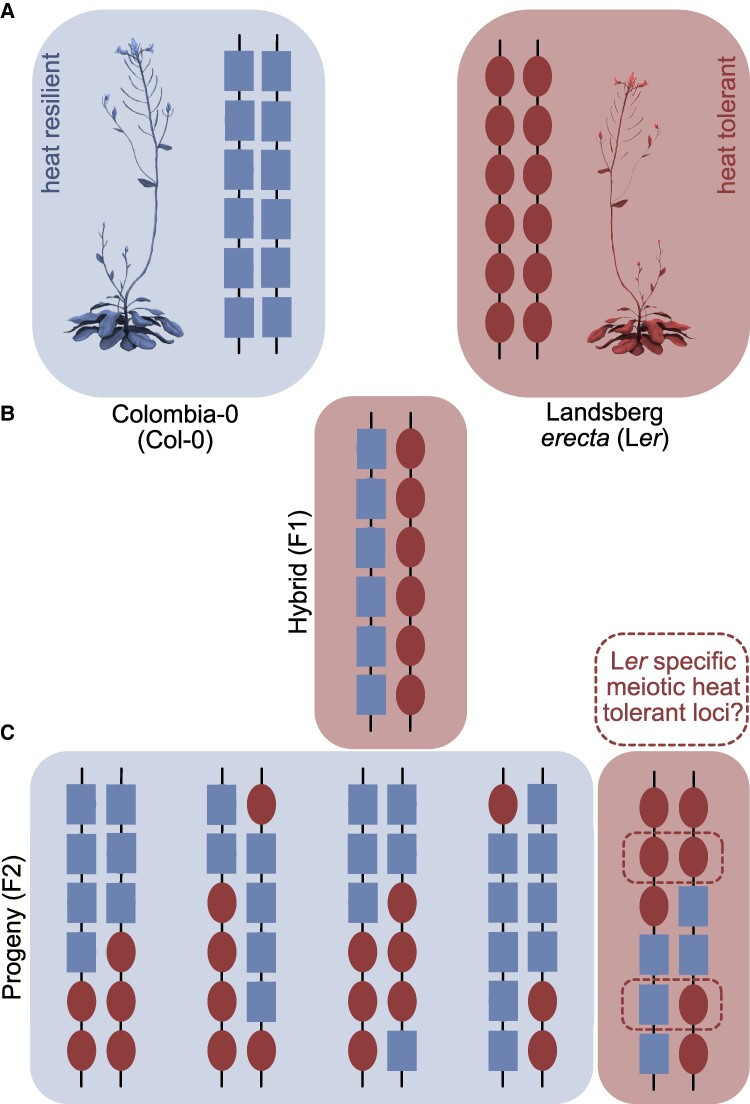
Schematic representation of thermoresilience and tolerance in Col-0 and L*er*. Parent plants Col-0 and Ler **(A)**, and their F1 hybrids **(B)** and F2 progeny **(C)**. One chromosome pair per individual plant shown. Blue squares represent Col-0 alleles and red ovals L*er* alleles. Blue boxes mark the heat resilient individuals and red boxes mark the thermotolerant individuals. Potential L*er* specific loci leading to thermos tolerance in dotted boxes. Arabidopsis drawing adopted from DataBase Center for Life Science (DBCLS), CC BY 4.0 via Wikimedia Commons.

The authors further tested if several kinases involved in floral architecture play a role in the heat response—for example, L*er* contains a mutation in kinase gene ERECTA (van Zanten et al. 2009). The mutants of those kinases in Col-0 had the same heat meiotic phenotype as Col-0, indicating that *ERECTA* is not the primary determinant of the heat tolerance.

Zhao et al. analyzed Col-0 and L*er* hybrids (F1) and their progeny (F2) under heat stress. Interestingly, although plant morphology of the hybrids was most similar to Col-0, the heat phenotype was more similar to L*er* than Col-0. This indicated that the L*er* thermotolerant loci are dominant over susceptible Col-0, which leads to the thermotolerant meiosis division in the hybrids (Fig. B).

Interestingly, when the authors tested the progeny of the Col/L*er* hybrids (the F2 plants) under heat, only 20% resembled the L*er* phenotype (Fig. C). This observation suggests that the heat tolerance of L*er* might be conferred by the cooccurrence of multiple L*er* alleles. In the L*er* parent and the F1 hybrids these alleles are present, but in the F2 population the Col-0 and L*er* alleles randomly recombined during meiosis, leading into progeny with different genetic background (Fig. C). As meiotic recombination causes allelic segregation that deviates from the canonical Mendelian segregation, it leads to a smaller proportion of the F2 population with the heat-tolerant phenotype.

To further obtain the L*er* specific genetic alleles that lead to thermotolerance during meiosis, it would be of great interest to obtain the genetic background of those heat-tolerant progeny. This might lead to a set of candidate genes that give rise to the thermotolerant phenotype of L*er* (Fig. C), which could be crucial for breeding purposes.

Overall, the research of Zhao and colleagues has contributed to our understanding of the effect of extreme heat stress in L*er* accession and Col-0/L*er* hybrids. These results pave the road for further research to identify the L*er* genetic loci that are responsible for the meiotic heat tolerance.

## Data Availability

No new data were generated or analysed in support of this research.
